# Editorial: Ano-rectal and gastro-esophageal cancer: diving into diagnostic and therapeutic imaging modalities for radiotherapy

**DOI:** 10.3389/fonc.2023.1276334

**Published:** 2023-08-31

**Authors:** Letizia Deantonio, Berardino De Bari, Pierfrancesco Franco

**Affiliations:** ^1^Radiotherapy Clinic, Oncology Institute of Southern Switzerland, Ente Ospedaliero Cantonale, Bellinzona, Switzerland; ^2^Faculty of Biomedical Sciences, University of Southern Switzerland, Lugano, Switzerland; ^3^Department of Radiation Oncology, Réseal Hospitalier Neuchâtelois, La-Chaux-de Fonds, Switzerland; ^4^Radiation Oncology Department, Azienda Ospedaliera Universitaria (AOU)‘Maggiore della Carità’, Novara, Italy; ^5^Department of Translational Medicine, University of Eastern Piedmont, Novara, Italy

**Keywords:** magnetic resonance imaging, PET/CT, cancer treatment, radiation therapy, radiomics

Morphologic and functional imaging applied to radiation therapy (RT) has a valuable role in both diagnostic and therapeutic setting. In recent years, there has been an increasing interest focused on imaging application as biomarker of tumor aggressiveness, treatment response, patients’ survival, and radiation-induced normal tissue toxicity aimed at better personalizing treatment approaches and potentially reducing acute and late effects, together with treatment burden.

This more extensive application of imaging in upper and lower gastro-intestinal tumor is intriguing considering the new treatment approaches evaluated in several clinical trials in this clinical setting. In rectal cancer, the new evidence in favor of total neoadjuvant therapy (TNT) and organ preservation strategies may replace the standard multimodality approach according to refined risk stratification. Moreover, the concept of wait and see in squamous cell esophageal cancer after neoadjuvant concurrent chemo-radiation achieving complete clinical response is under investigation.

In this Research Topic, nine articles were published, addressing recent advances in the use of imaging as potential marker of tumor aggressiveness and predictor of treatment response in patients with upper and lower gastro-intestinal cancer.

Few studies so far have explored novel diagnostic sequences for tumor staging and treatment response.

In gastric cancer, a study by Zhu et al. showed pretreatment dynamic contrast-enhanced magnetic resonance imaging (DCE-MRI) quantitative parameters and intravoxel incoherent motion diffusion-weighted images (IVIM-DWI) to predict for response to neoadjuvant treatment and help in recurrence free survival patients’ stratification.

In the era of TNT and organ preservation strategy for rectal cancer, MR imaging has a crucial role in the therapeutic assessment both staging and treatment response in order to optimize treatment strategy. In this Research Topic, Chen et al. evaluated a particular MR sequence, amide proton transfer weighted (APTw) MRI, combined with diffusion-weighted imaging (DWI) in predicting pathological complete response (pCR) in a series of 53 locally advanced rectal cancer (LARC). Pre-APT combined with pre-DWI achieved a good diagnostic performance in predicting good response to neoadjuvant treatment (AUC 0.89). APTw MRI was also analyzed by Li et al. showed that APT helped to assess rectal cancer prognostic factors, including tumor grade, histopathological type, and extramural vascular invasion (EMVI) status, but not primary tumor (T) and lymph nodes (N) status. Similarly, to predict tumor aggressiveness Hu et al. explored the significance of collagen examined *in vivo* based on rectal tomoelastography quantified stiffness and by histologically measured collagen volume fraction (CVF). Tomoelastography is a technique based on multifrequency MR elastography with diagnostic power as shown in other tumors. The overexpression of collagen was correlated with increased tumor stiffness and high risk of tumor aggressiveness. MR elastography seemed to add diagnostic value to MRI.

Another diagnostic technique was explored for primary tumor staging of gastric cancer. In several situation, tumor identification and staging is challenging and standard imaging techniques may not be able to detect the tumor, while others may mislead accurate staging. A meta-analysis by Zhang et al. evaluated the diagnostic performance of an innovative modality, a double-enhanced ultrasonography (DCEUS), for clinical T staging in gastric cancer. The findings by the 8 studies included were promising, however, still requiring confirmation before considering DCEUS in routine clinical practice.

There has been increasing interest in radiomics as new image-based markers that can predict survival outcome to personalize treatment strategy.

One meta-analysis (Deantonio et al.) showed the promising performance of 18F-flourodeoxyglucose positron emission tomography/computed tomography (18F-FDG PET/CT)-based radiomics models in predicting pCR following neoadjuvant chemoradiation in esophageal cancer (AUC 0.81, 95% CI: 0.74-0.9). Because the evidence is based on few retrospective and monocentric studies often using in-house software, the Authors highlighted the importance of planning clinical trials with a well-designed radiomics analysis.

Of a potential great interest and worthy of further studies is the Italian hypothesis-generating study by Di Dio et al. that explored the potential of radiomics elaborating a predictive model to support oncologists in deciding which drug to prescribe between oxaliplatin-based regimen and 5-fluorouracil or capecitabine regimen for neoadjuvant treatment of LARC. The radiomics analysis of T2-weighted (T2-w) MRI seems able to predict the probability of disease-free survival (DFS) discriminating those patients who can benefit from oxaliplatin-based regimen.

A study by Wang et al. focused on the value of dual-energy CT radiomics and showed that a radiomics model combined with longest short-axis parameter may become an effective biomarker for assessing lymph node metastasis in rectal cancer.

Furthermore, Ye et al. conducted an interesting study investigating deep learning-based tumor volume delineation in esophageal cancer. Gross tumor volume (GTV) delineation is an essential task in RT planning and require efforts, expertise, and time because it is in many cases a manual process. For esophageal cancer, GTV delineation is often highly variable and radiation oncologists need contrast-enhanced CT, FDG-PET/CT and esophago-gastric endoscopy information for an accurate identification. The authors developed and validated a two- streamed three-dimensional deep learning GTV segmentation model using the planning CT or planning CT and FDG-PET/CT. The performance of the two-streamed models was better for cT3 and cT4 primary tumor improving contouring accuracy with a reduction of inter-observer variation by 37% and contouring time with an average of 48%. The deep learning methods for delineation of target volumes are interestingly studying and may be potentially clinically relevant in this crucial and time-consuming medical task also in the era of adaptive RT.

In summary, the studies published in this Research Topic explored the capability of peculiar MR sequences and radiomics features derived from MRI, CT, and 18F-FDG PET in detecting tumor aggressiveness and assessing treatment response. The primary aim was to provide new knowledge and possibilities for a tailored treatment approach in this clinical setting.

Functional imaging analysis along with radiomics need to be upfront integrated in prospective multicentric study design to achieve robust clinical results and support further evidence to enrich clinical practice.

## Author contributions

LD: Conceptualization, Writing – original draft. BB: Conceptualization, Writing – review & editing. PF: Conceptualization, Writing – review & editing.

